# An Empirical Study on the Effect of Training Data Perturbations on Neural Network Robustness

**DOI:** 10.3390/s24154874

**Published:** 2024-07-26

**Authors:** Jie Wang, Zili Wu, Minyan Lu, Jun Ai

**Affiliations:** 1 The Key Laboratory on Reliability and Environment Engineering Technology, School of Reliability and Systems Engineering, Beihang University, Beijing 100191, China; 2 CRRC Zhuzhou Institute Co., Ltd., Zhuzhou 412001, China

**Keywords:** robustness, perturbation, adversarial training, convolutional neural network, empirical study

## Abstract

The vulnerability of modern neural networks to random noise and deliberate attacks has raised concerns about their robustness, particularly as they are increasingly utilized in safety- and security-critical applications. Although recent research efforts were made to enhance robustness through retraining with adversarial examples or employing data augmentation techniques, a comprehensive investigation into the effects of training data perturbations on model robustness remains lacking. This paper presents the first extensive empirical study investigating the influence of data perturbations during model retraining. The experimental analysis focuses on both random and adversarial robustness, following established practices in the field of robustness analysis. Various types of perturbations in different aspects of the dataset are explored, including input, label, and sampling distribution. Single-factor and multi-factor experiments are conducted to assess individual perturbations and their combinations. The findings provide insights into constructing high-quality training datasets for optimizing robustness and recommend the appropriate degree of training set perturbations that balance robustness and correctness, and contribute to understanding model robustness in deep learning and offer practical guidance for enhancing model performance through perturbed retraining, promoting the development of more reliable and trustworthy deep learning systems for safety-critical applications.

## 1. Introduction

The superior performance of Large Language Models in natural language processing tasks enables artificial intelligence (AI) to approach human behavior, and AI products have been deeply involved in all aspects of production and daily life. Artificial intelligence is increasingly being used in safety-critical domains such as self-driving cars, medical diagnosis, and malware detection. This has drawn growing attention to the trustworthiness of AI applications, aiming to ensure their safety, reliability, and controllability, thereby enhancing confidence when using AI. Many real-world cases, such as the Tesla driving accident and the ambivalent outputs of ChatGPT-3.5, have shown the inherent vulnerability of modern neural networks commonly used in AI. In fact, deep neural networks are vulnerable to various forms of threats, such as adversarial attacks [1,2,3], as well as environmental disturbances and noise [4,5], resulting in erroneous outputs and even leading to safety incidents.

The discovery of adversarial examples for image classification models in 2014 [1] made people aware of the flaws and vulnerabilities of deep neural network algorithms. In safety-critical applications, such potential risk is a matter of serious concern. As a result, robustness against adversarial attacks has been a major focus of deep learning, and a large number of attack and defense techniques have been proposed successively. Adversarial training [6,7,8,9], which means training with adversarial examples, is generally recognized in research and practice as one of the most effective defense strategies [10] and has been the most studied method in recent years. However, the drawbacks of adversarial training are also quite obvious; that is, the cost of such methods should not be neglected, including collecting data and training adversarially on data multiple times larger than original datasets [10]. Therefore, it is important for adversarial training to select appropriate adversarial examples and to determine the level of adversarial perturbation during iterative training, as they are essential to ensure successful retraining and improve the robustness of the model.

Besides robustness against adversarial attacks, other studies [4,5,11,12,13,14,15] have focused on the robustness of data noise. Similar to adversarial training, the technique of noisy training, which involves iteratively training models with noise added to the input of training samples, has shown promise in enhancing the robustness of models against noisy inputs. However, the effectiveness of noisy training also relies on the careful configuration of perturbed examples, noise levels, and noise types within the training dataset. In addition, other regularization methods that enhance the generalization capability of the model, such as dataset augmentation noise in output targets (for example, label smoothing and disturb label [16]), may also have a positive impact on the robustness of the model.

Incorporating perturbations into the training data has proven to be advantageous in enhancing model robustness. A comprehensive understanding of the relationship between training set perturbations and the resulting impact on model robustness is essential for effectively configuring perturbation training. In this regard, some studies have analyzed overfitting in adversarial training and pointed out that overfitting to the training set does, in fact, harm robust performance to a very large degree in adversarial robust training [17]. Bastani et al. [18] also pointed out that an improved model trained with adversarial samples will overfit the algorithm that produced the adversarial samples; that is, a robust model for one adversarial attack algorithm will not be effective against another adversarial attack algorithm. Jiang et al. [19] investigated the generalization of deep neural networks trained on noisy labels across different noise levels, noise types, network architectures, and training settings. Bekker et al. [20] proposed a training method for neural networks on data with unreliable labels. Existing studies show that regularization methods, such as adversarial training, are not always beneficial for model robustness. It is necessary to investigate the typical perturbations on training data to determine the necessary perturbation types, levels, and quantities for the robustness requirements of the model. In our previous work [21,22,23], the impact of adversarial attacks such as DeepFool [2], FGSM [3], and PGD [6] on the adversarial robustness of the convolutional neural network (CNN) models are analyzed, and some empirical conclusions for the adversarial training are given.

To get a comprehensive understanding of training data perturbations and model robustness for high-quality perturbation training, we aim to provide an empirical study on both qualitative and quantitative relationships between possible perturbations in the training dataset and robustness of the obtained model, including adversarial attack and noise on input images, disturbance on labels, and the sampling distribution of training dataset. To achieve this goal, the Fast Gradient Sign Method (FGSM) [3] algorithm is a suitable choice for adversarial attacks because it allows control over the level of perturbations. In the case of noise perturbations, Gaussian distributions are often used to model common noises in nature. Furthermore, analyses of the combined effects of multiple perturbation factors are conducted to investigate the impact of training with mixed perturbations on model robustness. Two types of multi-factor experiments have been conducted: dual-factor experiments involving combinations of two image perturbations, Gaussian noise and adversarial attack, and four-factor experiments including all four above-mentioned perturbations. So, we investigate the following research questions:

- RQ1: How does additive Gaussian noise on training images affect the robustness of CNN models?
- RQ2: How does FGSM adversarial attack on training images affect the robustness of CNN models?
- RQ3: How does class imbalance in the training set affect the robustness of CNN models?
- RQ4: How does the mislabeling of training examples affect the robustness of CNN models?
- RQ5: How does the robustness of CNN models change when there are both Gaussian and adversarial examples in the training set?
- RQ6: How does the robustness of CNN models change when all four aforementioned perturbations are combined?

The contributions of this paper are summarized into six findings, and their detailed evidences are explained in Section 3. These results provide valuable insights for robustness defense training and the practice of collecting high-quality training data.

### Related Work

Training data quality is one of the primary concerns in the field of artificial intelligence. Training with high-quality data that accounts for various scenarios and potential data perturbations can improve the model’s robustness. Many data-oriented regularization methods [24], for example, dataset augmentation, label smoothing and disturb label [16], dropout, and adversarial training, can be used to improve data quality and are beneficial for achieving high-performance, generalizable, and robust neural network models.

Adversarial training is a commonly used method to improve model robustness, especially adversarial robustness. Adversarial samples refer to inputs added with extremely small perturbations that are imperceptible to humans but can cause erroneous results with a high probability [1]. Applying various defense strategies such as adversarial detection [25,26,27,28], adversarial training [6,7,8,9], defensive distillation [29,30,31], randomization [32,33], and gradient masking [34,35,36] to the data, algorithms, training process, and testing process can nullify the adversarial attacks and thus improve the robustness in the adversarial attack environment. Adversarial training is a concise and efficient method. It augments training data with adversarial examples in each training loop and iteratively optimizes the network with the goal of minimizing the worst-case loss in the given perturbation region to improve the model performance in adversarial environments [10]. Adversarial training primarily focuses on enhancing the adversarial robustness of a neural network model. Within the research of neural network robustness, many assessment methods have been developed specifically to address adversarial attacks [37]. It is currently widely accepted that the adversarial robustness of neural networks is associated with the security property [15,37,38,39,40,41].

DIN SPEC 92001-2:2020 [15] defines corruption robustness (CR), i.e., robustness with respect to noisy signals or changes in the underlying data distribution. In real-world usage environments, the data input to the neural network often suffers from corruptions, such as Gaussian noise, signal degradation, geometric transformations of the image, etc., in which case the performance of the model is greatly reduced, meaning that it is not robust. The standard [15] considers CR to be a safety problem. This kind of robustness is also referred to as semantic robustness in some studies. Huang et al. [11] investigated the robustness of neural networks against random noises, such as image rotation and scaling. Mohapatra et al. [12] studied the problem of robustness verification against semantic perturbations such as hue, saturation, lightness, brightness, contrast, and rotation. Hendrycks and Dietterich [42] proposed the ImageNet-C dataset as a benchmark for testing data corruption perturbations. This dataset is used to evaluate the robustness of ImageNet classifiers and consists of 15 common image corruptions categorized into noise, blur, weather, and digital domain perturbations.

The rest of the paper is organized as follows: Section 2 presents the experimental design and study methodology utilized in this paper. Section 3 gives the answers to the six research questions. Section 4 reports the limitations of our work. Section 5 discusses some general conclusions drawn from the six findings of the experimental analyses. Finally, conclusions and future work are given in Section 6.

## 2. Experimental Design and Study Methodology

This section describes our methodology for investigating the impact of training perturbations on robustness, including the experimental subjects, robustness measurements, training perturbation settings, and methods for analyzing impact relationships.

### 2.1. Experimental Setup

The variation curves of robustness are analyzed on CNN models for the MNIST handwritten digit recognition task, and two architectures, LeNet-5 [43] and AlexNet-8 [44] are used. The MNIST dataset consists of handwritten digit images (0–9) and their corresponding labels. The images are grayscale and have a size of 28 × 28 pixels, with pixel values quantized to the range [0, 1]. The dataset comprises 60,000 training samples and 10,000 test samples. In the training set, the number of samples for each digit (0–9) is as follows: 5923, 6742, 5958, 6131, 5842, 5421, 5918, 6265, 5851, and 5949. In the test set, the number of samples for each digit (0–9) is as follows: 980, 1135, 1032, 1010, 982, 892, 958, 1028, 974, and 1009. The network architectures of LeNet-5 are the same as [43], and the MNIST images are resized to 32 × 32 to enable the LeNet-5. As the original AlexNet architecture in [44] is designed for RGB images with a size of 227 × 227, adjustments in the network layers and parameters are required to accommodate the 28 × 28 MNIST inputs. The AlexNet-8 used in this experiment has 5 convolutional layers and 3 fully connected layers.

The experiments were carried out on a machine with a dual-core CPU (Intel(R) Xeon(R) Silver 4214 CPU @ 2.20 GHz, Intel Co., Santa Clara, CA, USA), 64 GB of RAM, a GPU (NVIDIA GeForce RTX 2070 SUPER, NVIDIA Co., Ltd., Santa Clara, CA, USA) with 8 GB of VRAM, and the Ubuntu 20.04 LTS (Canonical Ltd., London, UK) operating system. The training process of the MNIST digit recognition model was developed based on the PyTorch framework. To eliminate other sources of uncertainty during the training process and ensure reproducibility of the results, this project fixed the loss function, optimizer, and other training parameters except for epochs. Additionally, random seeds for Python, PyTorch, and NumPy were fixed. In order to achieve optimal models for each training set, each model was trained until convergence, so the epoch parameter varies for each model. 

It is important to note that when investigating the impact of training data perturbations on the robustness of CNN models, efforts have been made to ensure that the variations are solely introduced through the data perturbations. Therefore, other factors that could influence the training results, such as the number of training samples, training order, and batch size, have been kept as much as possible. When organizing the perturbed training set, means such as replacing the original clean images with perturbed images were employed to maintain an equal number of samples in the training set.

### 2.2. Perturbation Measurements and Perturbed Training Setup

We conduct experiments to analyze the impact of attacks and/or corruptions, including Gaussian noise, adversarial noise, class imbalance in the training set, and label errors, on the robustness of CNN models. The perturbation of an input image is measured using the Euclidean distance in the pixel space ($l_{2}$ norm) between the perturbed image and the clean image. The class imbalance of the training set is measured by the ratio of the number of majority class to the number of minority class. The degree of label disturbance using the label error rate metric, which is the ratio of the number of samples with incorrect labels to the total number of samples in the dataset. By introducing different degrees of perturbations to the original clean training samples or the sampling distribution of the set, perturbed training sets with different levels can be obtained.

#### 2.2.1. Gaussian and Adversarial Training Sets for RQ1 and RQ2

A MNIST image is represented by a two-dimensional matrix where each element represents the brightness of the corresponding pixel. The degree of image perturbation is usually measured by the Euclidean distance ($l_{2}$ norm) between the two matrices before and after the perturbation, expressed as

(1) $$\begin{array}{c} d\left(x\right)={\left\|x-x^{\prime }\right\|}_{2}=\sqrt{\sum_{i}\sum_{j}{\left(x_{ij}-{x^{\prime }}_{ij}\right)}^{2}} \end{array}$$

where x is the clean image before perturbation, and $x^{\prime }$ is the perturbed image. In our experiments, both the Gaussian noise and the adversarial attack perturbations are measured by the $l_{2}$ norm. Distance metrics based on the $l_{p}$ norm are commonly used in the robustness analysis of neural networks, and conclusions derived under different norms can be extended to each other using norm inequalities [45,46]. The $l_{2}$ distance is a representative metric for robustness evaluation. Although there are several image similarity metrics available, such as cosine, keypoint, and histogram, which are theoretically applicable for measuring the degree of image perturbations, the $l_{2}$ distance is used to provide general conclusions since it is typically utilized in the robustness verification and evaluation studies. To construct a perturbed training set, a specific subset of samples is selected, and perturbed samples are generated by augmenting Gaussian noise or adversarial perturbations on the selected samples. By ensuring that each sample is subjected to the same $l_{2}$ distance of perturbation, the $l_{2}$ distance can be employed as a metric to quantify the level of perturbation in the training set. This approach facilitates the analysis of the relationship between image perturbation and model robustness, enabling a systematic examination of the variations observed in terms of image perturbation in training set and its impact on the model’s robustness. 

Gaussian noise is the most common type of noise that causes image distortion in the real world. Gaussian noise refers to a class of noise where the probability density function of the noise follows a Gaussian distribution, also known as a normal distribution. The Gaussian distribution has two parameters: the mean parameter μ and the standard deviation parameter σ. The probability density function of the Gaussian distribution is $f\left(x\right)=1/\sqrt{2\pi }\sigma exp\left(-{\left(x-\mu \right)}^{2}/2\sigma^{2}\right)$. Gaussian perturbed images can be obtained by adding noise with amplitudes following a Gaussian distribution to the brightness matrix of an image. In our experiment, we investigate the impact of Gaussian noise superimposed on the entire image. Thus, the mean parameter μ is set to 0, and the degree of Gaussian noise is controlled by varying the standard deviation parameter σ.

Among existing adversarial attack algorithms, the Fast Gradient Sign Method (FGSM) [3] is a white-box method based on gradient optimization that can successfully attack most neural networks. The FGSM algorithm is efficient and has the capability to generate adversarial samples of any desired levels of perturbation by adjusting the algorithm’s parameters. This convenience greatly facilitates the analysis of the impact patterns of training perturbations. The perturbations generated by FGSM are also additive to the original image. FGSM attack is based on the gradients of the model and is a model-specific attack method. The adversarial samples can be represented as $x^{\prime }=x+\varepsilon \cdot sign(\nabla_{x}J(\theta ,x,y))$, where θ is the parameters of a model, y is the label of input x, J(θ,x,y) is the cost used to train the neural network, and ε is the step parameter. Adversarial samples can be generated by moving a small step in the direction of the gradient of J(θ,x,y) which increases the value of the cost function to cause misclassification. Thus, ε represents the maximum allowable value of the perturbation and is used to control the perturbation degree. For a given model and a given original sample, the stride ε is proportional to the $l_{2}$ distance of an adversarial image.

To organize a Gaussian or FGSM perturbed training set with the desired level of perturbation, disturbed samples for that perturbation level are needed. Since the perturbation level is measured by the $l_{2}$ metric, and the parameter to adjust the magnitude of the Gaussian noise is the scale parameter σ, and for the FGSM attack that is the stride ε, we generate multi-level equidistant perturbed images measured by $l_{2}$ for a clean image by (1) firstly, generate a series of Gaussian or FGSM perturbed samples with different levels of perturbation by setting the values of σ or ε, (2) then select one sample from this set that is closest to the given $l_{2}$ distance as the perturbation sample for that particular $l_{2}$ distance, while ensuring that the error is within 1/4 of the interval between distances, (3) if there are no samples satisfying the error condition, fine-tune the parameters σ or ε and repeat steps (1) and (2) until the desired perturbed sample corresponding to the $l_{2}$ distance is obtained. Applying this procedure to each original clean sample will produce all desired perturbed samples with the $l_{2}$ distance and form the perturbed training set at that perturbation level. Irrelevant variables such as the number of image samples are kept equal in the experiments, i.e., the total number of training samples and the number of samples in each category are guaranteed to be the same for all perturbed training sets, eliminating the influence of irrelevant factors on the experimental results.

In the experiment, 24 perturbed training sets were generated with perturbation levels measured by the $l_{2}$ norm increasing at equal intervals. The original clean training set has a level of 0 in terms of Gaussian noise or FGSM adversarial perturbation. The perturbed training set was obtained by adding 2000 perturbed images of the same perturbation magnitude to each class in the clean training set. Therefore, the total number of samples in the perturbed training sets was 20,000 more than in the clean training set. The 24 perturbed training sets have perturbation magnitudes of 0.5, 1.0, 1.5, 2.0, 2.5, 3.0, 3.5, 4.0, 4.5, 5.0, 5.5, 6.0, 6.5, 7.0, 7.5, 8.0, 8.5, 9.0, 9.5, 10.0, 10.5, 11.0, 11.5, and 12.0 measured by $l_{2}$ norm. Each training set was used for two CNN architectures, LeNet-5 and AlexNet-8, to compare the effect of perturbation training on the robustness of different network architectures.

#### 2.2.2. Perturbed Training Sets with Class Imbalance for RQ3

The class imbalance problem is a perturbation in the distribution of data samples and is measured by the ratio of the number of samples in the minority class to the number of samples in the majority class. For multi-classification tasks, the imbalance degree of each class is measured by the ratio of the number of samples in each class to the number of samples in the majority class. The imbalance degree $CI\left(l\right)$ of class l can be calculated by

(2) $$\begin{array}{c} CI\left(l\right)=1-\frac{\left|\left\{x|\left(x,y\right)\in D,y=l\right\}\right|}{{max}_{k\in L}\left|\left\{x|\left(x,y\right)\in D,y=k\right\}\right|} \end{array}$$

where *D* denotes the training dataset, $\left(x,y\right)$ denotes a sample in the dataset, x is the input image and y is the label, $L=\left\{0,\ldots ,L\right\}$ denotes the set of classes predicted by the classification model. It is obvious that the larger the number of samples in the majority class, the smaller the ratio between the other classes and the majority class, indicating a higher degree of class imbalance. Conversely, when the dataset is more balanced, and the sample quantities of each class are closer, the ratio between non-majority classes and the majority class approaches 1, and the value of $CI\left(l\right)$ close to 0. Therefore, the range of $CI\left(l\right)$ is [0, 1], and a higher value of CI indicates a higher degree of class imbalance in the training dataset.

The degree of class imbalance in a dataset can be directly used to measure the degree of perturbation in that perturbed training set. For the multi-classification model in this experiment, the training set is set to have the same number of samples for all classes except the majority class, and the ratio of the number of samples for the minor class to that for the majority class is used to measure the perturbation degree of the imbalanced training set. Since the experiments were carried out on a public dataset and additional examples were not available, the class-imbalanced perturbed training set was obtained by reducing the number of samples in the non-majority classes. The perturbed training set constructed by this approach maintains the same number of samples in the majority class as the original set, while the number of samples in the remaining classes is determined based on the degree of class imbalance. By setting an equal number of samples for all minor classes, the imbalance measurement CI for each minor class is the same, which can be used to measure the perturbation degree of the dataset, facilitating the analysis of the impact of perturbations in the sampling distribution on the robustness of the model. The settings ensure that the number of majority class samples is consistent across different disturbed training sets to control the influence of irrelevant factors on the experimental results.

In the experiment, 8 perturbed training sets were generated with class imbalance degrees increasing at equal intervals. Since the original MNIST training set does not have an equal number of samples for each class, the imbalance rate of the original training set is represented by the imbalance degree of the class with the fewest samples, which is $CI\left(5\right)$ = 1 − 5421/6742 = 0.2. The perturbed training sets obtained by reducing the number of samples in the non-majority classes have the CIs of 0.25, 0.3, 0.35, 0.4, 0.45, 0.5, 0.55, and 0.6, respectively. Each training set was used to train two CNN architectures, LeNet-5 and AlexNet-8, to analyze the impact under different network structures, such as RQ1.

#### 2.2.3. Perturbed Training Sets with Incorrect Labels for RQ4

We also investigated the impact of label errors on model robustness. The label disturbance level was measured using the label error rate, which is the ratio of the number of samples with incorrect labels to the total number of samples in the dataset, denoted by

(3) $$\begin{array}{c} LD=\frac{\left|\left\{x|\left(x,y\right)\in D,\;y\neq y_{true}\right\}\right|}{\left|\left\{x|\left(x,y\right)\in D\right\}\right|} \end{array}$$

where *D* denotes the training dataset, $\left(x,y\right)$ is a sample and y is the label of image x, $y_{true}$ represents the ground truth of image x. A higher value of *LD* indicates a higher degree of label disturbance in the training dataset. In order to analyze the relationship between robustness and the magnitude of label disturbance, the experiment simplified the distribution of label perturbation by distributing them uniformly across the classes, where each class has the same number of samples with incorrect labels, such that the training set perturbation measurement is the class perturbation measurement. In addition, to avoid introducing changes in the distribution of labels, it is also necessary to maintain the original proportions of classes so that the new labels set for the perturbed samples in each class are uniformly distributed across the remaining classes. This allows the number of samples of each class in the perturbed training set to be the same as the original unperturbed set, and the total number of samples is also the same, eliminating the influence of irrelevant factors.

In the experiment, 10 perturbed training sets were generated, with label error rates increasing at equal intervals. The unperturbed clean training set is the original training set and has a label error rate of 0. The perturbed training set is obtained by replacing the original samples with perturbed samples, so the total number of samples is the same as the original unperturbed set. The labeling perturbation levels for the 10 perturbed training sets are 0.05, 0.1, 0.15, 0.2, 0.25, 0.3, 0.35, 0.4, 0.45, and 0.5, respectively. Similar to RQ1, each training set was used to train two CNN architectures, LeNet-5 and AlexNet-8.

#### 2.2.4. Perturbed Training Sets with Both Gaussian and Adversarial Examples forRQ5

The dual-factor experiment aims to analyze the combined effect of Gaussian noise and adversarial attack on model robustness. According to the image perturbation metric and the perturbed training set generation methods in Section 2.2.1, we created a total of 36 training sets by mixing two types of perturbations, considering all possible pairwise combinations of the perturbation levels: 0, 2, 4, 6, 8, and 10. To generate the perturbed training set at a specific perturbation level, we added 1000 images of each perturbation type, with the same perturbation magnitude, for each class. The result is that there are a total of 1000 images per class for each type of perturbation, and the perturbed training set has a total of 20,000 more samples compared to the clean training set, following the same configuration as in the single-factor experiments. The image perturbation measurement $l_{2}$ can serve as the perturbation level for the entire training set. Similar to RQ1, each training set was used to train two CNN architectures, LeNet-5 and AlexNet-8.

#### 2.2.5. Perturbed Training Sets with All Four Perturbations for RQ6

The four-factor experiment incorporates all four types of perturbations from the single-factor analysis into the training set to analyze the correlation between model robustness and the combined perturbations, which include disturbance on sampling distribution, input images, and labels. In the experiment, each type of perturbation was set with five levels. The $l_{2}$ values for the two types of image perturbation were 0, 2, 4, 6, and 8. The class imbalance metric CI took the values of 0.2, 0.25, 0.3, 0.35, and 0.4, and the label error rate LD took the values of 0, 0.05, 0.1, 0.15, and 0.2. Due to the limited experimental conditions, an orthogonal experimental design was used to address this four-factor with the five-level problem, 25 representative combinations were selected for the experiment, and the multi-correlation between model robustness and the four independent variables was analyzed.

### 2.3. Benchmark for Robustness Evaluation

To analyze the variation of robustness, it is necessary to quantitatively measure the robustness using an objective and standardized metric that allows for comparisons across different models. In our work, the impact of two types of input perturbations, adversarial attack, and Gaussian random noise, is investigated, so the robustness assessment benchmark primarily focuses on these two types of perturbations. Therefore, on the basis of the original test set, called MNIST-Test, we designed two additional test sets, MNIST-C and MNIST-A, for comprehensive and global robustness evaluation, following the approach of [42]. The two perturbed test sets are obtained by applying corruption and attacks to all samples in the original test set to various degrees. The robustness is measured using the model’s performance metrics on the test benchmarks. The MNIST-C set is used to measure model robustness against random noise, especially Gaussian noise, in our experiments. The MNIST-A set is used to measure the robustness against adversarial attacks and consists of adversarial examples generated using the FGSM algorithm.

In summary, the global robustness metrics of MNIST image classification models for impact analysis are as follows, including three test sets and corresponding robustness metrics, satisfy the robustness assessment of CNN models under real-world environments and deliberate attacks:

- MNIST-Test: clean test set, evaluate model performance in primary operational environments without perturbations; many studies consider it as a measure of the correctness property of the model;
- MNIST-C: random noise test set, evaluate robustness against random corruptions, especially Gaussian noise, called random robustness;
- MNIST-A: adversarial test set, evaluate robustness against adversarial attacks, especially FGSM attacks, called adversarial robustness.

The test accuracy is commonly employed as a metric of correctness and robustness. For the MNIST image recognition task, which involves classifying images into one of ten classes, the corresponding confusion matrix is shown in Table 1.

Accuracy is a metric that quantifies the proportion of correctly classified samples in relation to the total number of samples. In the case of a multi-class classification task such as MNIST, accuracy can be computed by summing the number of correctly classified samples in each class and dividing it by the total number of samples. This can be expressed formally as:

(4) $$\begin{array}{c} accuracy=\frac{\sum_{i=0}^{n-1}{TP}_{i}}{\sum_{i=0}^{n-1}{TP}_{i}+{FP}_{i}} \end{array}$$

where ${TP}_{i}={TP}_{ii}$ represents the true positive counts for class i, indicating the number of correct predictions, and ${FP}_{i}=\sum_{j=0,j\neq i}^{n-1}{FP}_{ji}$ represents the false positive counts, indicating the number of incorrect predictions. Since the MNIST dataset used in the experiments does not exhibit class imbalance and the robustness evaluation is more concerned with the model’s ability to give correct outputs for perturbed test inputs, employing accuracy as a metric to gauge the correctness of the model’s predictions is straightforward and representative. Thus, accuracy is an effective parameter for assessing robustness.

It is crucial to pay attention to the accuracy of the model on the original test set during the analysis of robustness impact factors. The accuracy of the original test set reflects the model’s recognition capability in a normal environment without perturbations. While enhancing robustness by injecting perturbations into the training data, it is necessary to ensure that the model’s predictions remain correct at a certain level. By maintaining a reasonable level of accuracy on the original test set, we can ensure that the model retains its ability to correctly classify samples in the common usage. This allows us to analyze the trade-off between robustness and correctness and make informed decisions about the model’s performance in different scenarios.

**Figure 1:**
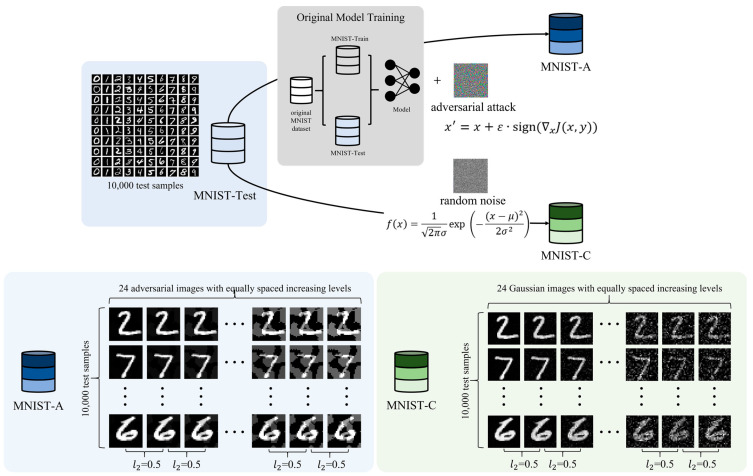
Robustness metrics and test set construction process for MNIST image classification models.

Figure 1 shows the robustness test set construction process. There are 10,000 test samples in the public MNIST-Test set, and we generated 24 levels of perturbations for each image, with equal increments. As a result, both MNIST-C and MNIST-A have 240,000 test samples each. The perturbation levels increase with an interval of 0.5 in terms of the $l_{2}$ distance, with the maximum perturbation level set to be 12.0 based on the criterion that it is distinguishable by the human eye. Each perturbation level has the same number of samples, which is 10,000 images. For each level, the sample count per class remains the same as the original test set (MNIST-Test). Specifically, there are 980, 1135, 1032, 1010, 982, 892, 958, 1028, 974, and 1009 samples for class 0–9, respectively.

### 2.4. Methodology for the Impact Relationships and Severities Analysis

The impact of training perturbations on the robustness can be represented by the changes in the accuracy of MNIST-C and MNIST-A with respect to the levels of perturbations in the training set. By plotting relevant tables and graphs, we can qualitatively describe the relationship between the two variables, and the quantitative relationship can be represented by the correlation coefficient (cc). For the single-factor impact analysis, the simple correlation coefficient, i.e., Pearson correlation coefficient, can be used to reflect the degree of association between two variables. The Pearson correlation coefficient is suitable for linear correlation analysis and has certain requirements for the variables, such as non-zero standard deviations for both variables and the normal distribution or an approximate unimodal distribution. Therefore, even if the Pearson correlation coefficient is 0, it only indicates the absence of a linear correlation, but a nonlinear correlation may still exist. When the variable distributions do not meet the linear requirements, the Spearman rank correlation coefficient is more practical, and if the coefficient is not significant, it indicates the absence of a correlation between the two variables.

The Spearman rank correlation coefficient is calculated as follows:

- Transform the raw data $X=\left[X_{1},X_{2},\ldots ,X_{n}\right],Y=\left[Y_{1},Y_{2},\ldots ,Y_{n}\right]$ into its sort number vector $x=\left[x_{1},x_{2},\ldots ,x_{n}\right],y=\left[y_{1},y_{2},\ldots ,y_{n}\right]$;
- The correlation coefficient is
  (5) $$\begin{array}{c} r=\rho \left(x,y\right)=\frac{cov\left(x,y\right)}{\sqrt{var\left(x\right)var\left(y\right)}}=\frac{\sum \left(x-\bar{x}\right)\left(y-\bar{y}\right)}{\sqrt{\sum {\left(x-\bar{x}\right)}^{2}}\sqrt{\sum {\left(y-\bar{y}\right)}^{2}}} \end{array}$$

The multi-factor impact analysis requires the multiple correlation coefficient to reflect the relationship among three or more variables. The multi-correlation coefficient is an extension of the simple correlation coefficient and is commonly used in multivariate linear regression analysis. When the dependent variable is affected by multiple independent variables collectively, the correlation between them is known as the multi-correlation coefficient, which reflects the degree of association between one variable and a set of other variables. To analyze the correlation between the dependent variable y and multiple independent variables X in practical problems, assuming a joint normal distribution between y and X and that there exists a linear correlation between them, the definition of the generalized multi-correlation coefficient is proposed, it is a natural extension of the simple correlation coefficient and the multiple correlation coefficient, and is more practical. The multi-correlation coefficient cannot be calculated directly, and the calculation method is as follows:

To determine the multi-correlation coefficient between the dependent variable y and multiple independent variables $X=\left(x_{1},x_{2},\ldots ,x_{k}\right)$, one approach is to construct a linear combination of $x_{1},x_{2},\ldots ,x_{k}$ and compute the simple correlation coefficient between this linear combination and y as the multiple correlation coefficient between y and $x_{1},x_{2},\ldots ,x_{k}$. The specific process is as follows:

- Constructing a linear model to regress y on $x_{1},x_{2},\ldots ,x_{k}$, get $\hat{y}=b_{0}+b_{1}x_{1}+\ldots +b_{k}x_{k}$;
- Correlation analysis of y with $x_{1},x_{2},\ldots ,x_{k}$ is the simple correlation analysis between y and $\hat{y}$, i.e.,:
  (6) $$\begin{array}{c} R=\rho \left(y,x_{1},x_{2},\ldots ,x_{k}\right)=\rho \left(y,\hat{y}\right)=\frac{cov\left(y,\hat{y}\right)}{\sqrt{var\left(y\right)var\left(\hat{y}\right)}} \end{array}$$

In general, the correspondence between correlation values (r or R) and correlation strengths is as follows:

- $\left|r\right|>0.95$: significant correlation;
- $0.8\leq \left|r\right|<0.95$: high correlation;
- $0.5\leq \left|r\right|<0.8$: moderate correlation;
- $0.3\leq \left|r\right|<0.5$: low correlation;
- $\left|r\right|<0.3$: weak or no correlation.

In addition to correlation coefficients, the experiment also analyzed the mean and variance of the accuracy of each model in robustness testing. These metrics describe the average performance level and overall variability of the models. Unlike statistical measures that describe the relationship between two variables, the mean and variance reflect the variation of a single variable. Since the perturbation increments are equally set, it is possible to analyze the average level of accuracy of the models on the robustness benchmark and the intensity of the change in accuracy as the training perturbations increase as a complement to correlation analysis. 

In summary, the relationships between training perturbation level and the robustness of CNN models involve:

1. Correlation diagrams: plot line graphs of robustness with increasing perturbation levels in order to analyze
  - the impact of training perturbations on the training fitting of CNN models;
  - the influence of training perturbations on the performance in the primary operational environment, whether it would lower the performance level;
  - whether CNN models trained with perturbations are robust when operating in the perturbed environment, and the direction of the effect, whether the robustness improves or deteriorates as the level of training perturbations increases.
2. Correlation coefficient:

Conduct a correlation analysis to examine the correlation coefficient between the magnitude of training perturbations and the robustness of CNN models. This helps understand the strength and direction of the relationship between these variables.

3. Mean and variance of accuracy:
  - Analyze whether the CNN models are robust when operating in a perturbed environment through mean accuracy, which provides insights into the overall performance;
  - Assess the variability or variance of the accuracy of CNN models through variance, which helps understand the extent to which the robustness changes with increasing perturbation.

## 3. Empirical Results and Findings

The performance and robustness of the models were evaluated on three test sets proposed in Section 2.3. The changes in robustness with increasing perturbations were depicted using line graphs. The correlation between training perturbation levels and model robustness was analyzed by the Spearman rank correlation coefficient, and the variability of model accuracy was used to indicate the degree of robustness variation. The single-factor correlations are calculated by the Scipy library in Python, and the multi-factor correlations are calculated by the multiple linear regression function lm in R.

### 3.1. RQ1: How Does Additive Gaussian Noise on Training Images Affect the Robustness of CNN Models

Finding #1: Gaussian noise training has a positive effect on model robustness. However, the original model already exhibits relatively good robustness in the random noise environment, so the impact is not significant. It also has limited effectiveness in improving the robustness against adversarial attacks and does not make the model adversarially robust. Therefore, the improvement in model robustness through Gaussian noise training is limited. Additionally, the effect of Gaussian noise training is dependent on the network architecture. The LeNet-5 network exhibits poor stability in Gaussian noise perturbation training, with the model’s training fitting greatly affected by the noise. The same hyperparameters as the original LeNet-5 model cause the perturbed model to be trapped in a local optimum, resulting in oscillations in robustness. It can be concluded that there are no universally applicable hyperparameter settings for the training process (e.g., batch size, optimizer, etc.). Adjusting hyperparameters requires more effort to achieve the optimal model in specific perturbation training. On the other hand, the AlexNet-8 demonstrates excellent stability in this regard. Therefore, the effectiveness of Gaussian noise training on model robustness is limited, and its impact varies depending on the network, as observed in the experimental results.

**Figure 2:**
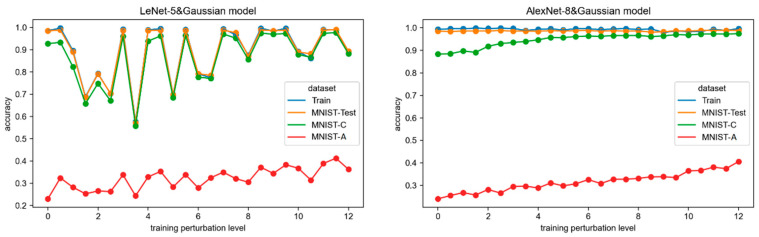
Variation of robustness under Gaussian noise training.

The LeNet-5 and AlexNet-8 networks were trained on 24 Gaussian noisy training sets and obtained 24 models for each (denoted as LeNet-5&Gaussian models and AlexNet-8&Gaussian models, respectively). The accuracy of each model on the training set and the three test sets are plotted in Figure 2 to observe the trend of accuracy with increasing Gaussian perturbation on the training data. From the line diagrams, we can derive some qualitative relationships between model robustness and the level of Gaussian noise perturbation during training.

Model fitting. The training set accuracy of the LeNet-5 model fluctuates significantly with the degree of Gaussian noise in the training set. This indicates that the training fitting of the LeNet-5 network is highly influenced by Gaussian noise. To ensure the validity of the analysis and minimize the influence of extraneous factors, all aspects were kept constant except for the level of perturbation. This included using identical hyperparameters, such as learning rate and batch size, during the training of both the perturbed model and the original model. This approach may lead the perturbed LeNet-5 model to be trapped in a local optimum. However, we intentionally avoid performing additional optimizations during training in order to accurately demonstrate the effect of data perturbations on the model. Therefore, determining suitable hyperparameters requires careful effort when training LeNet-5 models on Gaussian noise datasets. Interestingly, when the perturbation is relatively small (especially for perturbation levels of 1.5, 2.0, 2.5, 3.5, 5.0, 6.0, and 6.5), the training fitting is relatively poorer. On the other hand, as the Gaussian noise in the training set increases, the model tends to achieve better training fitting. In contrast, the training set accuracy of the AlexNet-8 model is consistently close to 1.0 for all perturbation levels, indicating that the training of the AlexNet-8 is not affected by Gaussian noise on the input.

Primary task performance. The accuracy of the MNIST-Test set reflects the model performance in the primary operational environment. Due to the influence of training fitting, the LeNet-5 models exhibit significant fluctuations in accuracy on the MNIST-Test. In contrast, the accuracy of the AlexNet-8 models is consistently close to 1.0. Therefore, the impact of Gaussian noise perturbation training is different depending on the network architecture and its fitting results. For LeNet-5, the presence of perturbations hinders them from achieving optimal fitting, resulting in degraded performance on the primary task. On the other hand, Gaussian noise perturbation training does not lower the performance level of the AlexNet-8 models in the primary operational environment.

Random robustness. Regarding the random robustness of the models indicated by the accuracy on MNIST-C, all LeNet-5 models trained with Gaussian noise have accuracies on MNIST-C that are not far from those on the original test set MNIST-Test. This indicates that the performance of LeNet-5 models in the random Gaussian noise environment is not weakened, and they are robust to this perturbation. From the results, it can be concluded that the Gaussian noise training will not make the random robustness of the LeNet-5 models aggravation. Besides, the accuracy of the AlexNet-8 models on MNIST-C shows a clear upward trend as the training perturbation increases. The accuracy rises from below 0.9 to a level close to 1.0. This indicates that the larger the Gaussian noise added during training, the better the performance of the AlexNet-8 models in a random noise environment. It can be inferred that the AlexNet-8 model is robust in a random noise environment regardless of the magnitude of Gaussian noise in the training examples. And Gaussian noise perturbation training can effectively enhance the random robustness of the AlexNet-8 network.

Adversarial robustness. Regarding the adversarial robustness of the models reflected by the accuracy on MNIST-A, the LeNet-5 models exhibit low and fluctuating accuracy on the MNIST-A test set, with none of them exceeding 0.4. However, there is an overall increasing trend in accuracy as the training perturbation level increases. Similarly, the accuracy of the AlexNet-8 models on MNIST-A also improves with larger training perturbations, but the accuracy levels do not surpass 0.4. Therefore, it can be drawn that the Gaussian noise in the training set has a slight positive effect on the model’s adversarial robustness, but the improvement is not significant. In fact, models trained with Gaussian perturbations do not exhibit strong robustness in adversarial environments.

From the changes in the accuracy of the LeNet-5 model on the three robustness test sets, it can be observed that the fluctuations in accuracy closely mirror the accuracy fluctuations in the training set accuracy. This phenomenon indicates that the model performance is heavily influenced by its training fitting. Gaussian noise in the training set affects the fitting of the model and, hence, its performance. This effect is particularly evident when the LeNet-5 model operates in the primary operational environment represented by MNIST-Test and the random noise environment represented by MNIST-C. On the other hand, the accuracy of the AlexNet-8 models shows no significant fluctuations in the three test sets because they consistently achieve good training fitting. This suggests that the AlexNet-8 network is less affected by the Gaussian noise in the training set and exhibits good stability.

The quantifiable relationship between the accuracy of the three test sets and the level of Gaussian noise in the training set of the two CNN networks is shown in Table 2. The Spearman rank correlation coefficient (*r*) between the test set accuracy and the Gaussian noise on input images measures the correlation strength, and the *p*-value from the significance test shows whether the correlation is significant or not, with *p* < 0.05 indicating a significant correlation between the two variables. Additionally, the mean and variance of the accuracy of all models were analyzed to reflect the average level and degree of variation in model performance or robustness under different training perturbation levels.

Correlation coefficient. Based on the values of *r*, it can be observed that the accuracy on all three test sets is positively correlated with Gaussian noise. According to the strength of the correlation provided in Section 2.4, only the performance of the AlexNet-8 model on the MNIST-C and MNIST-A test sets meets the criteria of moderate or high correlation (|*r*| ≥ 0.8) and significance (*p* < 0.05). This indicates a strong positive correlation between Gaussian noise training and the robustness (both random and adversarial) of the AlexNet-8 model. In other words, training with Gaussian noise perturbation can enhance the robustness of the AlexNet-8 model in both random noise and adversarial attack environments, with a greater effect seen with larger levels of Gaussian noise. On the other hand, due to the poor training fitting of many models of the LeNet-5 network, it is pointless to quantitatively analyze the correlation of the LeNet-5 models based on the available experimental data. It can be roughly concluded that Gaussian noise training is weakly correlated (|*r*| < 0.5) with the accuracy on MNIST-Test and MNIST-C for the LeNet-5 models, indicating a small improvement in their random robustness, and there is a moderate positive correlation (*r* = 0.73) with the accuracy on MNIST-A although the significance is low, which suggests a certain degree of improvement in the model’s robustness against adversarial attacks. These conclusions align with the qualitative conclusions drawn from the analysis based on Figure 2.

Mean and variance. From the mean and variance, it can be observed that the models of both networks achieve mean accuracies above 0.8 in the primary operational environment MNIST-Test and the random noise environment MNIST-C. The mean accuracy of the AlexNet-8 models even exceeds 0.9. However, in the adversarial attack environment MNIST-A, the mean accuracies of both networks are only around 0.3, indicating poor performance. This suggests that the models trained with Gaussian noise are robust in the presence of random noise, with the AlexNet-8 model exhibiting better robustness. However, they are not robust in adversarial attack environments. Regarding the variance of accuracy, except for the LeNet-5 models, which have relatively high variance in accuracy on MNIST-Test and MNIST-C, the variances of the other networks and test sets are small. This means that the performance of the LeNet-5 models is more sensitive to the level of Gaussian noise during the training process. However, considering the accuracy of the model on the training set, it can be inferred that the variations are mainly influenced by the fluctuation in the model’s training fitting. Although the robustness of the AlexNet-8 model is positively correlated with Gaussian perturbation, the improvement is limited. This is because the original unperturbed model already possesses a rather high level of random robustness, leaving little room for improvement. Additionally, Gaussian noise training does not make the model robust against adversarial attacks.

### 3.2. RQ2: How Does FGSM Adversarial Attack on Training Images Affect the Robustness of CNN Models

Finding #2: Adversarial perturbation training has a significant positive effect on the adversarial robustness of the model and can maintain a high level of random robustness. A certain degree of adversarial training does not affect the model’s performance in the primary operational environment or the robustness in the presence of random noise. Instead, it significantly improves the model’s robustness in adversarial attack environments, making the model exhibit good levels of robustness in both random noise and adversarial attack environments. However, when the adversarial perturbations in the training set are too large, it will negatively impact the fitting performance of the LeNet-5 network, leading to a decrease in both model performance and robustness. Additionally, the robustness of the AlexNet-8 model in random noise environments may also deteriorate, and its robustness in adversarial attack environments may decrease again. Empirically, it is observed that including adversarial perturbations in the training data should not exceed 8 (using the $l_{2}$ metric). Furthermore, the LeNet-5 network exhibits poorer stability under adversarial training.

**Figure 3:**
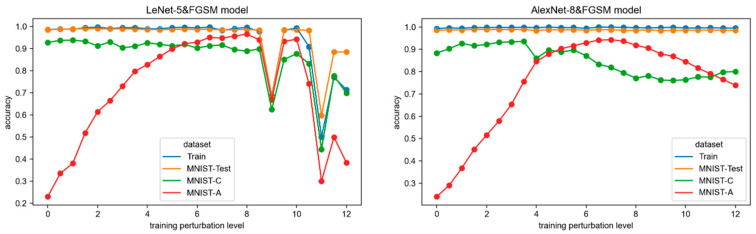
Variation of robustness under adversarial training.

The two CNN networks were trained on 24 FGSM adversarial training sets to obtain 24 LeNet-5&FGSM and AlexNet-8&FGSM models, respectively. Figure 3 shows the accuracy of the models on the training set (Train) and three test sets (MNIST-Test, MNIST-C, and MNIST-A) under different perturbation levels in adversarial training. As can be seen:

Model fitting. The training set accuracies of the LeNet-5 models are all close to 1 when the adversarial perturbation on the training set is small. However, when the adversarial perturbation level increases to 9.0, the accuracies exhibit significant fluctuations. The accuracies of the LeNet-5 models are below 0.7 for adversarial perturbations of 9.0 and 11.0, indicating an unacceptable fitting result. On the other hand, the training set accuracy of the AlexNet-8 model consistently remains close to 1.0. Therefore, we can conclude that within a certain range, the adversarial training does not affect the model’s fitting result. However, excessive adversarial perturbations on the training set can negatively impact the fitting of the LeNet-5 network, indicating poorer stability under adversarial training.

Primary task performance. The accuracy of the MNIST-Test reflects the performance of the models on the primary tasks. The performance of the LeNet-5 model is influenced by the training fitting. When the adversarial perturbations on the training set are below 9.0, the accuracy on the MNIST-Test can remain close to 1.0. However, as the perturbations increase to 9.0, the accuracy starts fluctuating in accordance with the training set accuracy. On the other hand, the accuracy of the MNIST-Test for the AlexNet-8 model remains consistently close to 1.0. It can be concluded that adversarial training does not decrease the performance of the CNN models in the primary operational environment. However, excessive perturbations can affect the fitting results and consequently impact the model’s performance.

Random robustness. The accuracy of MNIST-C reveals the robustness of the models in a random noise environment. The accuracy of the LeNet-5 models shows slight variations around 0.9 when the adversarial perturbations are below 9.0; as the perturbations increase to 9.0, the accuracy decreases and exhibits significant fluctuations due to fitting issues during training. On the other hand, the MNIST-C accuracy of the AlexNet-8 models tends to decrease with increasing adversarial perturbations. With perturbation levels less than 3.5, the accuracy has a high level and is able to improve, whereas the random noise environment accuracy starts to decrease when the adversarial perturbations continue to increase but still maintain more than 0.8. These observations suggest that the models under adversarial training still exhibit robustness in random noise environments. However, adversarial training does lead to a certain degree of reduction in the models’ random robustness.

Adversarial robustness. Regarding the model robustness in an adversarial environment reflected by the accuracy on MNIST-A, both networks show a trend of initially increasing and then decreasing accuracy as the adversarial perturbations on the training set increase. The accuracy of the LeNet-5 models starts decreasing after the perturbation level reaches 8.0, while for the AlexNet-8 model, it occurs after 7.0. The decrease in MNIST-A accuracy for the LeNet-5 model aligns with the fluctuations in training fitting. Regardless of the fitting results, adversarial robustness decreases when the training perturbation is too large, indicating that adversarial training with excessive perturbation has a negative impact on the model’s robustness in adversarial environments. From the experimental data, for the LeNet-5 network, a range of adversarial perturbation levels within [4.0, 8.5] enables the model to maintain an adversarial robustness above 0.8. The corresponding range for the AlexNet-8 model with an adversarial robustness above 0.8 is [4.0, 10.5]. Therefore, appropriate adversarial training can enhance the models’ adversarial robustness significantly.

Table 3 shows the Spearman rank correlation coefficients *r* and *p*-values between the adversarial perturbation level and the accuracy of the models on three test sets. The mean and variance of the accuracy are also displayed. From Figure 3, it can be observed that the changes in model robustness exhibit a clear nonlinear relationship, particularly in regard to adversarial robustness on MNIST-A. Although the Spearman rank correlation coefficient can analyze the nonlinear relationship between two variables, in order to better measure the strength of the correlation between the two variables, the analysis divides the training perturbation levels into two parts based on the increasing and decreasing stages of MNIST-A accuracy. The correlation coefficients, means, and variances of the accuracy on each test set are calculated separately for these two segments. The two perturbation ranges for LeNet-5 are as follows: Segment I [0.0, 8.0] and Segment II [8.5, 12.0]. For AlexNet-8, the ranges are Segment I [0.0, 7.0] and Segment II [7.5, 12.0].

Correlation coefficient. As can be seen from the correlation coefficient *r*, the MNIST-Test and MNIST-C accuracies of both networks are negatively correlated with the adversarial perturbation levels on the training set in Segment I. According to the correlation strength discussed in Section 2.4, the correlation between MNIST-Test accuracy and FGSM perturbations is low (|*r*| < 0.5) and not statistically significant. The correlation on MNIST-C is slightly stronger but still only moderately correlated. The accuracy of MNIST-A is significantly positively correlated (*r* > 0.95) with the adversarial perturbation levels. In Segment II, except for the significantly negative correlation (*r* = −1.0) between MNIST-A accuracy and adversarial perturbation levels for the AlexNet-8 model, the accuracies on the other test sets do not exhibit a significant correlation with the perturbation levels as *p* > 0.05. Based on these results, it can be concluded that there is no correlation between adversarial training and the performance of the models in the primary task environment and a weak negative correlation with the models’ random robustness but a significant correlation with the models’ adversarial robustness, and when the training perturbations are relatively small, it can significantly improve the models’ adversarial robustness. However, excessive perturbations lead to a rapid decrease in the models’ adversarial robustness.

Mean and variance. It can be observed that in Segment I, the mean accuracies of the MNIST-Test and MNIST-C for both networks are around 0.9, with the performances on the MNIST-Test even approaching 1.0, and the variances of these accuracies are small. However, the mean accuracies of both networks on MNIST-A are relatively lower, and it has larger variances, indicating a significant variation in adversarial robustness. This suggests that a certain level of adversarial training does not affect the models’ original performance and random robustness but has a significant impact on the models’ adversarial robustness. In Segment II, the LeNet-5 model exhibits large variances in the accuracies on all three test sets. Considering the fluctuation of the model’s accuracy on the training set, it can be inferred that the significant variation in robustness is primarily influenced by the training fitting. The mean accuracies of MNIST-Test and MNIST-A for the AlexNet-8 models remain above 0.8, while it is relatively lower on MNIST-C. This indicates that excessive adversarial training can decrease the model’s random robustness; from the amplitude indicated by the variances, the impact seems to be not substantial.

### 3.3. RQ3: How Does Class Imbalance in the Training Set Affect the Robustness of CNN Models

Finding #3: Although the class imbalance perturbations in the training set do not affect the performance of CNN models in the primary operational environment, they are not effective in enhancing the models’ robustness in adversarial environments. In fact, they may even decrease the models’ robustness to random noise environments. Therefore, class imbalance perturbations of the training data sampling distribution have minor effects on model robustness, so the robustness cannot be improved by adjusting or optimizing the training data sampling distribution.

**Figure 4:**
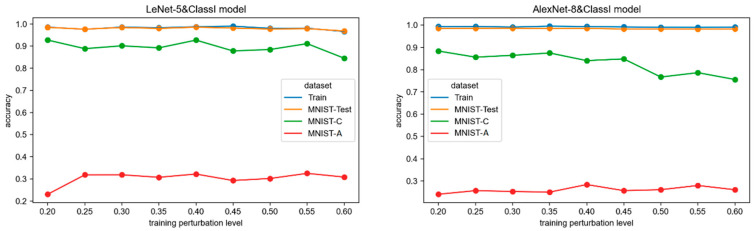
Variation of robustness under class imbalance training.

The accuracy trends of the two CNN network models (LeNet-5&ClassI and AlexNet-8&ClassI models) trained on the 8 perturbed training sets of class imbalance are shown in Figure 4. As can be seen:

Model fitting. The training accuracies of all models are close to 1.0, except for the LeNet-5 network, which shows a slight decrease in training accuracy when the class imbalance rate is 0.6. This indicates that the models can still achieve good fitting even in the presence of this type of perturbation during training. Only when the class imbalance issue is severe will it slightly affect the training fitting of the model.

Primary task performance. As the degree of class imbalance in the training set worsens, the accuracy of all models on the original test set, MNIST-Test, remains essentially unchanged, and the models perform well. Only the LeNet-5 model at a class imbalance rate of 0.6 shows a decrease in accuracy on the MNIST-Test due to the decrease in training fitting. Despite the presence of a class imbalance in the training set, the model’s performance in the primary operational environment does not change much, suggesting that the distribution of training samples has a minor effect on the model performance.

Random and adversarial robustness. The robustness of the model in perturbed operational environments, as reflected by its accuracy on two perturbed test sets, is indeed influenced by the class imbalance issue in the training set. From the line chart, it can be observed that class imbalance in the training set tends to decrease the accuracy of the model in the random noisy environment, MNIST-C while showing a slight improvement in accuracy in the adversarial environment, MNIST-A. However, since the accuracies on MNIST-A are very low, around 0.3, the improvement in adversarial robustness due to class imbalance is not substantial. Overall, models trained with class imbalance perturbations exhibit lower accuracy in perturbed environments, and the impact of class imbalance on model robustness is negative.

Table 4 shows the Spearman rank correlation coefficients *r* and *p*-values between test accuracy and class imbalance degree, as well as the mean and variance of the accuracy for each network. The quantitative impact is also analyzed in terms of the degree of correlation and the overall change.

Correlation coefficient. From the values of the Spearman rank correlation coefficient and their significance, it can be observed that the accuracy of MNIST-Test and MNIST-C is negatively correlated with class imbalance, while the accuracy of MNIST-A is positively correlated. The *p*-values for all three test sets of the LeNet-5 network do not meet the requirement of being less than 0.05, indicating that the correlation is not significant, while those of AlexNet-8 are all less than 0.05. According to Section 2.4, the strength of the correlation coefficient on the MNIST-Test and MNIST-A is moderate, while it is high on the MNIST-C. In summary, the class imbalance disturbance on the training set is not strongly correlated with the robustness of the CNN models, but it may have a negative impact on the models’ random robustness.

Mean and variance. The average accuracy of the two CNN networks on MNIST-Test and MNIST-C can reach over 0.8. However, the average accuracy on MNIST-A is only around 0.3, indicating a low performance level in an adversarial attack environment. This suggests that class imbalance has a positive impact on training models to be robust in random noise environments but lacks robustness in adversarial attack environments. Both networks have low variances in accuracy on all test sets, indicating that the change in accuracy is small. This further suggests that the impact of class imbalance disturbance on model robustness is weak.

### 3.4. RQ4: How Does the Mislabeling of Training Examples Affect the Robustness of CNN Models

Finding #4: Mislabeling the training examples can affect the optimization and the fitting of the model, thereby affecting its performance in the primary operational environment. It also reduces the random robustness to some extent. Although adversarial robustness is increasing, the practice of implanting incorrect labels in training examples is still insufficient to make the model robust against adversarial attacks. In summary, the impact of label perturbation on model performance and robustness is negative, and the disturb label approach as a regularization method to prevent overfitting does not improve the model’s robustness.

**Figure 5:**
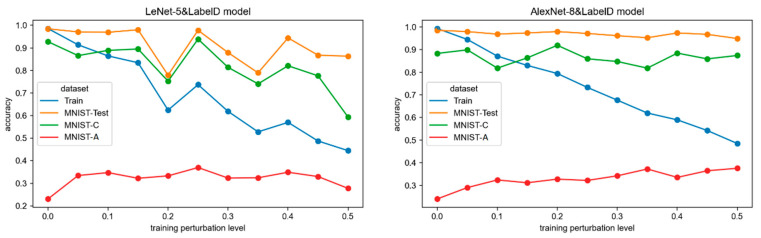
Variation of robustness when training with disturbed labels.

The accuracy trends of the two CNN network models (LeNet-5&LabelD and AlexNet-8&LabelD models) trained on the 10 disturb label training sets are shown in Figure 5. As can be seen:

Model fitting. As the label perturbation in the training set increases, the training accuracy of both network models sharply decreases. Since accuracy is a metric based on the difference between labels and predictions, the incorrect labels make the measurements meaningless. So, in this experiment, clean test set accuracy is used to monitor the model optimization process.

Primary task performance. The LeNet-5 model achieves a stable accuracy close to 1.0 on the MNIST-Test when the label error rate is less than 0.15. As the perturbation level continues to increase, the accuracy starts to fluctuate, but all above 0.8. The MNIST-Test accuracy of the AlexNet-8 mode can reach close to 1.0 at any perturbation level. This indicates that the issue of label errors in the training samples does not significantly affect the model’s fitting and performance in the primary operational environment.

Random robustness. The LeNet-5 model shows fluctuations in accuracy on MNIST-C as the label error rate increases, and overall, it exhibits a noticeable decrease. However, the accuracy remains above 0.7 for most models (except the perturbation level at 0.5). The AlexNet-8 model demonstrates smaller variations in accuracy on MNIST-C, and around 0.9. Therefore, different networks exhibit varying robustness in a random noise environment. The impact of label perturbation on the random robustness of the AlexNet-8 model is minimal, and it possesses high robustness to random noise. The random robustness of LeNet-5 models decreases with increasing label perturbation, and it exhibits poor random robustness when the perturbation exceeds 0.15.

Adversarial robustness. All models exhibit low accuracy on MNIST-A. Although there is an increasing trend in accuracy with the growth of training perturbation, it remains below 0.4. This indicates that the label perturbation in the training set cannot make the models robust in adversarial environments.

Table 5 shows the Spearman rank correlation coefficients *r* and *p*-values between test accuracy and label perturbation degree, as well as the mean and variance of the accuracy for each network. The quantitative impact is also analyzed in terms of the degree of correlation and the overall change.

Correlation coefficient. It can be observed from the values of *r* and *p* that the label error rate in the training set is negatively correlated with the accuracy of MNIST-Test and MNIST-C and is positively correlated with the accuracy of MNIST-A. Additionally, according to Section 2.4, the correlation strength is moderate and significant for LeNet-5 models on MNIST-Test and MNIST-C, as well as for the AlexNet-8 models on MNIST-Test. Only the correlation for the AlexNet-8 models on MNIST-A is high and significant, while the rest show weak correlations. In summary, the correlation between the disturb label in the training set and the robustness of CNN models is not strong.

Mean and variance. The mean accuracy of both networks on MNIST-Test and MNIST-C is above 0.8, while the mean accuracy on MNIST-A is only 0.3. This suggests that the models trained with disturb labels are robust in the presence of random noise but lack robustness in adversarial attack environments. Both networks exhibit small variances in the accuracy of all test sets, indicating minimal variation in the accuracy of perturbed modes. This further proves that the impact of label perturbation in the training set on model robustness is weak. This phenomenon is similar to that observed in class imbalanced training.

### 3.5. RQ5: How Does the Robustness of CNN Models Change When There Are Both Gaussian and Adversarial Examples in the Training Set

Finding #5: The combined effect of two types of input image perturbations on the training examples has a significant positive impact on the model’s adversarial robustness. The linear correlation between random robustness and the perturbation level is dependent on the network architecture. Furthermore, the presence of both image perturbations in training does not severely degrade the model’s performance in the primary operational environment.

**Figure 6:**
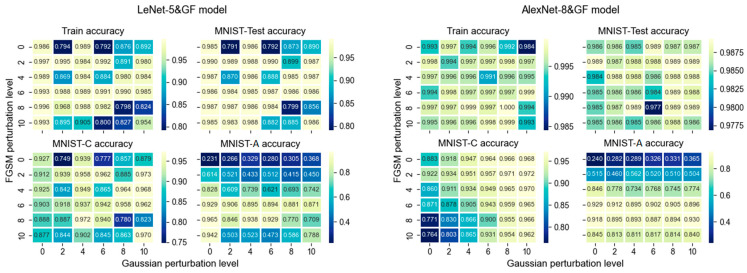
Variation of robustness under dual-factor perturbation training.

The accuracy trends of the two CNN network models (LeNet-5&GF and AlexNet-8&GF models) trained on the 26 combined perturbation training sets of Gaussian noise and FGSM attack are shown in Figure 6. As can be seen:

Model fitting. Analyzing the training accuracy of the LeNet-5 models under the same level of adversarial perturbation, it fluctuates or decreases significantly with the increase of Gaussian noise level, especially when the adversarial perturbation level is high (level of 8 and 10); the training accuracy drops to around 0.8, and when the adversarial perturbation level is 6, it is less affected by Gaussian noise. This indicates that the fitting result of the LeNet-5 network is negatively affected by Gaussian noise under the dual-factor combined perturbations. On the other hand, the training accuracies of the AlexNet-8 models are all close to 1.0, indicating that the training fitting is little affected by the dual-factor perturbations.

Primary task performance. The accuracy of the LeNet-5 model on the MNIST-Test fluctuates in sync with the training accuracy. When only one of the perturbations is high, the model can maintain an accuracy close to 1.0. When the adversarial perturbation remains small, and the Gaussian noise is increased, the accuracy significantly decreases, reaching a minimum of 0.79. When the level of Gaussian noise is 2, the model can maintain a high accuracy regardless of the level of adversarial perturbation. Therefore, under dual-factor perturbations, the performance of the LeNet-5 models in the primary operational environment can be affected by poor model fitting, and to maintain accuracy at a certain level, the Gaussian noise level should not exceed 6. On the other hand, the AlexNet-8 model achieves an accuracy close to 1.0 on the MNIST-Test, with a minimum of 0.97. Therefore, the dual-factor input perturbation does not lower the performance of the AlexNet-8 model on the primary tasks.

Random robustness. All models of both CNN networks under different combinations of perturbation levels achieve an MNIST-C accuracy of at least 0.75. For the AlexNet-8 model, under the same level of adversarial perturbation, increasing Gaussian noise improves the model’s random robustness, while under the same level of Gaussian perturbation, increasing adversarial perturbation decreases the random robustness. Models above the main diagonal can achieve a random robustness of over 0.9, while models below the diagonal exhibit lower random robustness, with a minimum of 0.76. The model trained on the clean training set already possesses a certain level of random robustness, so perturbation training may reduce the random robustness. It can be argued that some combinations of dual-factor perturbation training can enhance the random robustness. The adversarial perturbation level should not exceed 6, and it is unnecessary to introduce excessive Gaussian noise. Going beyond these limits may lead to diminishing returns or even negatively impact the model’s random robustness.

Adversarial robustness. It is only when the adversarial perturbation level is increased to 4 in the dual-factor perturbation training that both CNN network models start to achieve an accuracy of over 0.6 on the MNIST-A. However, as the adversarial perturbation is increased to 8, the accuracy of LeNet-5 models begins to decline. On the other hand, under the same level of adversarial perturbation, the models’ adversarial robustness does not show significant variation with changes in Gaussian noise. Therefore, the models trained on the clean training set are not robust in an adversarial environment, and the dual-factor perturbation training can effectively enhance the adversarial robustness, with an appropriate range for adversarial perturbation level being 6 to 8. The magnitude of Gaussian noise is irrelevant.

Table 6 presents the bivariate correlation coefficients and significance levels between the accuracy of the two CNN network models on the three test sets and the two independent perturbation variables, adversarial perturbation level and Gaussian perturbation level. Based on the correlation strength provided in Section 2.4, the MNIST-Test accuracy of both CNN networks and the MNIST-C accuracy of LeNet-5 show a weak and non-significant correlation with dual-factor perturbation training, while the MNIST-A accuracy of LeNet-5 shows a moderate and significant correlation. Both MNIST-C and MNIST-A accuracies of AlexNet-8 demonstrate a strong and significant correlation with dual-factor perturbation training. These results indicate that the combined effect of the two image perturbations has a positive impact on the models’ adversarial robustness. The linear correlation between perturbation levels and random robustness depends on the network architecture. The performance variation of the models in the primary operational environment does not follow a clear linear pattern.

### 3.6. RQ6: How Does the Robustness of CNN Models Change When All Four Perturbations Are Combined

Finding #6: The combined perturbation training with four kinds of perturbations at the same time may have a linear positive correlation with the adversarial robustness of the model, which can improve the adversarial robustness of the model without reducing the level of random robustness.

Table 7 presents the multiple Pearson correlation coefficients and their significance between the accuracy of two CNN network models on the three test sets and the four types of perturbations in the training set. Based on Section 2.4, the accuracy of the LeNet-5 model on MNIST-Test and MNIST-C shows a low and insignificant correlation with the four-factor perturbation training, while the accuracy on MNIST-A exhibits a high and significant correlation. For the AlexNet-8 model, the correlation of accuracy on MNIST-Test is significant, while those of MNIST-C and MNIST-A are moderate. These results indicate that under the combined four perturbations, the performance of the LeNet-5 models on the primary tasks and its random robustness does not exhibit a clear linear pattern with training perturbation, but the adversarial robustness shows a strong linear relationship. On the other hand, the AlexNet-8 models yield different conclusions, as the combined four training set perturbations significantly impact the model’s performance in the primary operational environment, but the robustness, including random and adversarial robustness, does not show a clear linear correlation.

According to the experimental results, it can be concluded that for the LeNet-5 network, there is no obvious linear pattern of random robustness in the perturbation training with the combined four types of disturbances, but there exists a strong linear relationship with adversarial robustness. For the AlexNet-8 model, this combined perturbation training improves its performance in the primary operational environment, but the linear relationship with robustness is not strong, which suggests that the AlexNet-8 model may behave differently for different types of perturbations or the impact of combined perturbations on the robustness may not follow a simple linear relationship.

## 4. Threats to Validity

Similar to other empirical studies, our study is naturally subject to validity problems. We identify potential threats to the validity of our study as follows:

Threats to Conclusion validity. Our conclusions are only for CNN models performing image classification tasks, and the experimental results may not be representative of the overall population of CNN networks and datasets. This threatens the generalizability of our findings in real-world scenarios beyond the specific networks and datasets studied. We address this limitation by conducting experiments on multiple networks and giving specific conclusions for different networks, but further research on a broader range of models is needed for stronger generalization.

Threats to Construct validity. We analyze the impact of four distinct perturbation types on model robustness, but when considering perturbations related to sampling distribution and labeling errors, it becomes challenging to implant them on the test set, thereby capturing the robustness against these perturbations. Our analysis primarily focuses on two fundamental aspects: random robustness and adversarial robustness. This approach aligns with the usual practice in the field of robustness assessment.

Threats to Internal validity. The performance of a trained model can be influenced by various factors, including training parameters, sample size, and the training order of samples. These factors can impact the model’s fitting results and its subsequent robustness. Consequently, the robustness measurements are not solely determined by the perturbations introduced in the training data but also by the intricacies of the training process. To mitigate the impact of irrelevant variables on the training results, we conducted our experiments by keeping other unrelated variables fixed across all models and ensuring consistent training conditions across experiments. These settings allow us to better analyze the specific impacts of perturbations.

Threats to External validity. One potential threat to the external validity of our study lies in the magnitude of perturbations used, particularly in the case of Gaussian and adversarial disturbances. The FGSM attack algorithm used in our experiment may be too large to be discernible by the human eye, making the evaluation of robustness and subsequent impact analysis potentially meaningless. We mitigated this threat by constraining the perturbation levels within a range where the human eye can determine the classification of the image. This approach strengthens the validity of our findings and enhances the applicability of our conclusions in real-world scenarios where human perception plays a crucial role.

## 5. Discussion

We perform a comprehensive empirical study on the factors affecting neural network robustness. Through this study, we found: (1) the perturbation training with Gaussian noise and adversarial attack on the input image has a positive effect on model robustness, while class imbalance perturbation and labeling perturbation in the training set have a negative but weak effect on model robustness. (2) the practice of adding Gaussian noise to the training set provides a limited improvement in both random robustness and adversarial robustness, as the original clean model already has relatively high random robustness, whereas the adversarial training can significantly improve the model’s adversarial robustness and maintain the original random robustness. Therefore, the optimal approach for enhancing model robustness is adversarial perturbation training, as it positively influences the model’s resistance to adversarial attacks and preserves its existing resilience to random noise.

Single-factor perturbation case. Training a model with a specific data perturbation can only improve the robustness of the model in the corresponding perturbation environment. For example, Gaussian perturbation training is effective in enhancing the model’s robustness against Gaussian noise but does not make the model robust to adversarial attacks. On the other hand, adversarial training significantly improves the model’s adversarial robustness but may sacrifice its resilience to random noise. Moreover, to enhance the overall robustness of the model, adversarial training is most necessary, but the magnitude of adversarial perturbations added to the training set should be carefully controlled. It is suggested that the perturbation level measured by $l_{2}$-norm should not exceed 6 when using the FGSM algorithm to implement adversarial attacks based on this empirical experiment.

Multi-factor perturbations case. The multi-correlation between model robustness and a set of perturbation variables can only be assessed through multiple linear regression analysis to determine the degree of linear correlation. In general, there is a high linear correlation between the combination of multiple perturbations and the model’s adversarial robustness, indicating that increasing the perturbation level in the training set can enhance the model’s adversarial robustness. However, its effect on random robustness depends on the network architecture. It is important to note that increasing the intensity of multi-factor perturbation training may significantly affect the model’s performance in the primary operational environment, meaning there might be a trade-off between correctness and robustness in combined perturbation training. Besides, if there is no linear relationship between robustness and a set of perturbations, it is possible that they have some form of non-linear relationship. This would require more advanced analytical methods and finer-grained data support.

In addition, the model performance is closely related to the training accuracy, both on the original test set and the three perturbed test sets. Therefore, improving the training fitting has a positive impact on its robustness. However, not all perturbed training sets can achieve good fitting results. For example, training sets with Gaussian noise or disturb labels may not lead to optimal fitting. Additionally, the LeNet-5 network exhibits poor stability, as the training fitting fluctuates significantly with increasing perturbation levels on the training set. This makes it challenging to determine the optimal perturbation level when designing perturbed training sets.

## 6. Conclusions and Future Work

In this paper, we investigate the impact of perturbations in training data on the robustness of models. We explore various forms of perturbations that can occur in different aspects of the dataset, including input, label, and sampling distribution. Our research encompassed both single-factor and multi-factor perturbation training experiments, which were carried out on two CNN architectures. The experimental results yield six useful findings that can provide guidance for constructing high-quality perturbation training datasets aimed at optimizing model robustness and achieving enhanced performance. By adding appropriate perturbations for model training, meaningful patterns can be learned, enabling better generalization to unseen data in the primary environment and improving robustness in the presence of perturbed inputs. It is important to keep perturbation levels within reasonable limits to ensure the balance between model robustness and accuracy across diverse environments. These findings will be invaluable in the development of more effective strategies for constructing training datasets that facilitate the optimization of model performance in real-world applications. However, the influence mechanism that connects data perturbations with the resulting robustness of trained models is complex. This relationship is contingent upon factors such as the architecture and scale of the network. Due to limitations in computational resources, we only performed empirical studies on the simplest publicly available image dataset and two networks adapted to it. In the future, we intend to investigate larger datasets and a broader range of network architectures to draw more comprehensive and generalizable conclusions.

## Figures and Tables

**Table 1:** Confusion matrix of MNIST image recognition task.

		Predicted				
		Class 0	Class 1	Class 2	…	Class 9
Actual Class	Class 0	TP_00_	FP_01_	FP_02_	…	FP_09_
	Class 1	FP_10_	TP_11_	FP_12_	…	FP_19_
	Class 2	FP_20_	FP_21_	TP_22_		FP_29_
	…	…	…	…	…	…
	Class 9	FP_90_	FP_91_	FP_92_	…	TP_99_

**Table 2:** Quantitative relationship between Gaussian noise and model robustness.

Test Dataset	LeNet-5				AlexNet-8			
	*r*	*p*	Mean	Variance	*r*	*p*	Mean	Variance
MNIST-Test	0.275	0.184	0.891	0.0147	0.408	0.043	0.986	3.45 × 10^−6^
MNIST-C	0.489	0.013	0.866	0.0144	0.977	5.83 × 10^−17^	0.947	8.42 × 10^−4^
MNIST-A	0.730	3.347	0.322	0.0023	0.984	7.24 × 10^−19^	0.315	0.0017

**Table 3:** Quantitative relationship between adversarial perturbations and model robustness.

Test Dataset	LeNet-5							
	Segment I				Segment II			
	*r*	*p*	Mean	Variance	*r*	*p*	Mean	Variance
MNIST-Test	−0.460	0.063	0.987	2.97 × 10^−6^	−0.357	0.385	0.872	0.0204
MNIST-C	−0.716	0.001	0.917	1.92 × 10^−4^	−0.500	0.207	0.750	0.0210
MNIST-A	0.998	9.58 × 10^−19^	0.737	0.0546	−0.690	0.058	0.676	0.0585
**Test Dataset**	**AlexNet-8**							
	**Segment I**				**Segment II**			
	***r***	***p***	**mean**	**variance**	***r***	***p***	**mean**	**variance**
MNIST-Test	−0.063	0.825	0.987	2.44 × 10^−6^	0.268	0.454	0.985	8.32 × 10^−7^
MNIST-C	−0.557	0.031	0.894	0.0012	0.345	0.328	0.779	2.03 × 10^−4^
MNIST-A	1.0	0.0	0.681	0.0607	−1.0	6.65 × 10^−64^	0.846	0.0041

**Table 4:** Quantitative relationship between class imbalance and model robustness.

Test Dataset	LeNet-5				AlexNet-8			
	*r*	*p*	Mean	Variance	*r*	*p*	Mean	Variance
MNIST-Test	−0.483	0.187	0.980	2.46 × 10^−5^	−0.795	0.010	0.984	2.71 × 10^−6^
MNIST-C	−0.533	0.139	0.895	5.88 × 10^−4^	−0.900	0.0009	0.831	0.0021
MNIST-A	0.250	0.516	0.303	7.43 × 10^−4^	0.717	0.030	0.260	1.69 × 10^−4^

**Table 5:** Quantitative relationship between label error rate and model robustness.

Test Dataset	LeNet-5				AlexNet-8			
	*r*	*p*	Mean	Variance	*r*	*p*	Mean	Variance
MNIST-Test	−0.645	0.032	0.910	0.0055	−0.755	0.007	0.970	1.15 × 10^−4^
MNIST-C	−0.682	0.021	0.820	0.0093	−0.236	0.484	0.866	8.73 × 10^−4^
MNIST-A	0.009	0.979	0.322	0.0013	0.909	0.0001	0.328	0.0014

**Table 6:** Quantitative impact of dual-factor perturbations.

Test Dataset	LeNet-5		AlexNet-8	
	*r*	*p*	*r*	*p*
MNIST-Test	0.322	0.163	0.361	0.100
MNIST-C	0.105	0.832	0.892	4.39 × 10^−12^
MNIST-A	0.653	1.00 × 10^−4^	0.850	6.38 × 10^−10^

**Table 7:** Quantitative impact of four-factor perturbations.

Test Dataset	LeNet-5		AlexNet-8	
	*r*	*p*	*r*	*p*
MNIST-Test	0.414	0.415	0.964	3.15 × 10^−11^
MNIST-C	0.373	0.535	0.779	6.15 × 10^−4^
MNIST-A	0.922	5.75 × 10^−8^	0.529	0.143

## Data Availability

The data presented in this study are available in the PyTroch library at torchvision.datasets.MNIST.
